# Advances in positron emission tomography and radiomics

**DOI:** 10.1002/hon.3137

**Published:** 2023-06-09

**Authors:** Sally F. Barrington

**Affiliations:** ^1^ School of Biomedical Engineering and Imaging Sciences St Thomas' Campus, Kings College London London UK

**Keywords:** diffuse large B cell lymphoma, lymphoma, positron emission tomography, radiomics

## Abstract

Positron emission tomography is established for staging and response evaluation in lymphoma using visual evaluation and semi‐quantitative analysis. Radiomic analysis involving quantitative imaging features at baseline, such as metabolic tumor volume and markers of disease dissemination and changes in the standardized uptake value during treatment are emerging as powerful biomarkers. The combination of radiomic features with clinical risk factors and genomic analysis offers the potential to improve clinical risk prediction. This review discusses the state of current knowledge, progress toward standardization of tumor delineation for radiomic analysis and argues that radiomic features, molecular markers and circulating tumor DNA should be included in clinical trial designs to enable the development of baseline and dynamic risk scores that could further advance the field to facilitate testing of novel treatments and personalized therapy in aggressive lymphomas.

## 1 WHAT IS RADIOMICS?

Radiomics is the extraction of quantitative features from standard of care patient scans, in contrast to simple visual interpretation. Radiomic analysis offers the potential for data mining of multiple image features, which may be related to pathophysiology in patients with cancer that can serve as biomarkers to assist with clinical decision making.^1^, ^2^, ^3^ This approach is analogous to genomic analysis, employing bioinformatics to prioritize key features that can be used in clinical prediction models, differentiating them from those that are highly correlated and likely to be redundant.

Models combining complementary clinical, imaging and genomic information are now being developed in lymphoma.^4^, ^5^ An advantage of radiomic compared to genomic analysis is that positron emission tomography‐computed tomography (PET‐CT) imaging is routinely performed throughout the course of treatment in most lymphomas and standardized imaging approaches have already been widely adopted.^6^

Radiomics is typically considered to comprise high throughput extraction of comprehensive quantitative information, for example, about the texture and shape of lesions, which may be hidden to the human eye.^2^, ^3^ In this article however, simple “first‐order” imaging features will mainly be discussed, as these “conventional” PET metrics have been reported to be prognostic, with more complex textural features performing less well.^7^, ^8^ Baseline total metabolic tumor volume (tMTV),^9^, ^10^, ^11^, ^12^, ^13^, ^14^, ^15^, ^16^, ^17^, ^18^ tumor heterogeneity^19^, ^20^ and disease dissemination markers^21^, ^22^ are associated with prognosis and the standardized uptake value (SUV) of FDG‐avid lesions^23^, ^24^, ^25^, ^26^, ^27^ is known to be associated with therapy response in lymphoma entities.

Methods for segmentation are key for the extraction of quantitative tumor metrics, with various delineation methods reported so far.^28^ These different methods have resulted in different optimal cut‐offs being proposed for separating patients into prognostic groups using tMTV,^10^, ^28^ which has probably held back the field. Progress is now being made toward standardized methods for measurement of tMTV in lymphoma.^28^ Since different methods are associated with similar prognosis,^29^, ^30^, ^31^ the preferred measurement method is one that is reproducible, requires minimal observer interaction and is suitable for use in busy clinical practice,^32^ adding no more than 5 min to a clinical report in most cases.^28^ The “MTV4” method which automatically segments areas with SUV ≥4.0 required the least manual editing when tested against other commonly applied methods in diffuse large B‐cell lymphoma (DLBCL)^32^ and Hodgkin lymphoma (HL)^33^ and was the least sensitive to the influence of different imaging reconstructions from different PET systems.^34^ This method is gaining acceptance amongst researchers.^35^, ^36^, ^37^ A minimum volume of lesions to be included in an automated approximation of tMTV can be specified within software programmes (e.g., 3mls)^32^ which simplifies measurement of tMTV further with little impact on the overall volume estimation.

A benchmark dataset is under construction by an international consortium applying the MTV4 measurement method using three different imaging softwares in patients with HL, DLBCL and follicular lymphoma.^28^ The benchmark dataset is intended to allow end users including PET readers and software manufacturers to test their ability to measure tMTV within a predefined range and as a reference against which to examine potential improvements such as deep learning approaches for automatic segmentation, which are likely to be the future.^38^, ^39^, ^40^, ^41^ This will make a significant difference to the field if widely adopted.

An option to pool results obtained using different measurement methods or imaging protocols exists called the COMBAT method.^2^, ^42^ This is a statistical tool which realigns data that have different distributions, subject to certain assumptions, enabling harmonization of image derived features.^2^, ^42^ COMBAT can be used for retrospective analysis of clinical trial imaging data, with a minimum of at least 20–30 patients to align data from different datasets.^42^

## 2 BASELINE RISK PREDICTION IN LYMPHOMA

Multiple studies from different research groups have reported the prognostic value of tMTV using ^18^Fluorine‐fluorodeoxyglucose (FDG) PET‐CT in the first line treatment of HL,^12^, ^16^, ^17^, ^43^ DLBCL^11^, ^13^, ^24^, ^44^ FL^15^ and peripheral T‐cell lymphoma,^9^ in the relapsed/refractory setting^45^, ^46^ and in patients receiving CAR‐T cell therapy.^47^, ^48^, ^49^, ^50^ Tumor lesion glycolysis (TLG) (tMTV × mean SUV) is also prognostic but with the exception of primary mediastinal B‐cell lymphoma (PMBCL)^51^ which typically presents with large dominant mediastinal masses, the prognostic value appears similar for tMTV and TLG. As TLG is more complex and introduces another potential source of variability by using SUV and tMTV,^28^ most research groups have focused on tMTV for estimation of tumor burden. Whilst tMTV appears to be a robust biomarker, criticism has been leveled at earlier studies due to their retrospective nature and because many lack external validation.^52^ Most studies have relied on the median value or on the receiver operating characteristic analysis to define the optimal cut‐offs for prognostic groups, usually “high” and “low” MTV. This gives results that are highly dependent on the study population in which they are derived and risks overestimating the value of tMTV.^52^ How best to combine tMTV with other surrogates for tumor burden used in established clinical risk scores has also been unclear.

Recently a model was developed incorporating baseline MTV4 with components of the international prognostic index (IPI) in an international cohort of 1241 patients with newly diagnosed DLBCL treated with rituximab, cyclophosphamide, doxorubicin, vincristine and prednisone (R‐CHOP) from five clinical trials.^35^ The optimal model for prediction of 3‐year progression‐free‐survival (PFS) and 3‐year overall survival (OS) included three components of age, Ann Arbor stage (I‐IV) and tMTV expressed as continuous variables. The statistical relationship between tMTV and survival was not linear, with increments at lower values having a greater adverse impact than at higher values of MTV. The relationship was best described using a linear spline with different coefficients applied above and below the median. The model proved to be robust, as the optimal model when four studies were combined to create a test set and externally validated in a fifth independent dataset. This exercise was repeated five times using a “leave one out approach,” validating the age‐stage‐MTV model each time. The proposed international “metabolic” prognostic index (IMPI) outperformed the IPI and defined a high‐risk group comprising 10% of the population with 3‐year PFS 46.3% versus 58.0% and 3‐year OS 51.5% versus 66.4% for IMPI and IPI, respectively. The use of continuous variables rather than dichotomous cut‐offs has the added advantage of allowing individual patient estimates of survival or to choose clinically relevant cut‐offs for testing treatment approaches in clinical trials (Table 1).

**TABLE 1:** Summary of relevant studies reporting baseline radiomic analysis in clinical trials.

Author	Number and patient population/trial	Median FU	Disease	Treatment	Model	Method of measurement and cut‐offs as applicable	Statistical relationship with survival	PFS/OS	Comments
Ceriani et al.^19^	103	62 months	PMBCL first line I‐IV	R‐CHOP R‐CHOP‐like R‐VACOP‐B R‐MACOP‐B	MTV + metabolic Heterogeneity (MH)	MTV of hottest lesion using SUV ≥2.5 and AUC‐CSH for MH cut–off 0.45	ROC‐AUC analysis	5 years PFS 94% versus 73% in low and high MH groups, respectively (*p* = 0.0001)	
	IELSG 26								
Ceriani et al.^20^	141 test	64 months	DLBCL first line I‐IV	R‐CHOP	MTV + MH	MTV of hottest lesion using SUV ≥2.5 and AUC‐CSH for MH Cut‐off 931 cm^3^ PFS, 1149 cm^3^ OS 0.43 MH	ROC‐AUC analysis	5 years PFS 83% versus 61% (*p* = 0.0005), 5 years OS 91% versus 65% (*p* = 0.0001), in low and high MTV respectively	In high MTV group, pts with high MH had higher risk of progression (HR, 5.6; 95% CI, 1.8–17) and death (HR, 9.5; 95% CI, 1.7–52)
	113 validation SAKK 38/07 study								
Cottereau et al.^21^	95	44 months	DLBCL first line I‐IV 60–80	R‐CHOP R‐ACVBP	tMTV + Dmax	tMTV using 41% SUVmax Cut‐off >394 cm^3^ SDmax >58 cm	ROC‐AUC analysis	4 years PFS 94% versus 73% versus 53% and 4 years OS 97% versus 88% versus 50% for pts with 0, 1 or 2 risk factors (*p* = 0.0003 PFS, *p* = 0.0011 OS) respectively	
	LNH073B								
Cottereau et al.^22^	290	5 years	DLBCL first line I‐IV 60–80	R‐CHOP	tMTV + SDmax (Dmax standardized by body surface area)	tMTV using 41% SUVmax Cut‐off 220 cm^3^ SDmax >0.32 m^−1^	ROC‐AUC analysis	4 years PFS 90% versus 63% versus 41% and 4 years OS 95% versus 79% versus 66% for pts with 0, 1 or 2 risk factors (*p* = 0.0001) respectively	
	REMARC								
Driessen et al.^27^	65	40 months	HL R/R	BV‐DHAP	sTARC + TLR using SUV peak lesion + SUV mean liver and sTARC after one cycle 1 of BV‐DHAP and SUVpeak prior to ASCT	sTARC cut‐off 500 pg/mL baseline TLR ≥3.0 pre‐ASCT TLR ≥1.0 using liver SUVmean and lesion SUVpeak	ROC‐AUC analysis	3 years FFP 35% versus 95% for pts with high baseline sTARC and high baseline TLR versus either high risk factor 3 years FFP 0% versus 95% for 4 pts with high sTARC post c1 and high TLR pre‐ASCT versus all pts	
	BRAVE								
Durmo et al.^53^	155 retrospective RW	63 months	HL I‐IV	ABVD	Dmax + MTV	Median Dmax 20 cm	Median used to subdivide pts	4 years PFS 90% versus 72% for pts with low and high Dmax respectively	Gene expression and cell populations differentially expressed in high and low Dmax groups
Eertink et al.^8^	317	92 months (parent trial)	DLBCL first line I‐IV	R‐CHOP	Radiomics (MTV4, SUVpeak Dmaxbulk) clinical (IPI components and bulk >10 cm)	tMTV4 Continuous scale	ROC‐AUC analysis	2 years TTP, 28% versus 44% for high risk pts for radiomics + clinical model and IPI respectively	Patient level features performed better than individual lesion analysis Simple radiomics model performed better than complex model
	HOVON‐84								
Eertink et al.^4^	323		DLBCL first line I‐IV		Radiomics (MTV4, Dmaxbulk, DSUVpeak, spread) + MYC status	tMTV4 Continuous scale	ROC‐AUC analysis	2 years TTP 50% versus 70% 2 years PFS 51% versus 65% 2 years OS 57% versus 69% for high risk pts for radiomics + MYC model and IPI respectively	
	HOVON‐84 PETAL								
Mikhaeel et al.^35^	1241	55 months	DLBCL first line I‐IV	R‐CHOP	MTV‐age‐stage	tMTV4 using SUV ≥4.0 and 3mls min volume Continuous scale	Linear spline with 2 coefficients above and below median 310 cm^3^	3 years PFS for high risk pts 46.3% versus 58.0% and 3 years OS 51.5% versus 66.4% for IMPI and IPI respectively	Split by scale into low risk 60% intermediate risk 30% high risk 10%
	5 clinical trials from PETRA consortium								
Thieblemont et al.^54^	1825 PETAL + GOYA	47.1–76.5 m	DLBCL first line I‐IV	R‐CHOP	MTV + PS	tMTV using 41% SUVmax (PETAL, RW) and liver threshold (GOYA) PS ≥2	COMBAT method used to harmonize tMTV measurements	4 years PFS 54% versus 59% (PETAL) 49% versus 58% (GOYA) 36% versus 55% (RW) for high risk pts for MTV + PS and IPI respectively *p* < 0.001 4 years OS 61% versus 70% (PETAL) 61% versus 72% (GOYA) 41% versus 59% (RW) for high risk pts for MTV + PS and IPI respectively *p* < 0.001	
	349 RW								
Vercellino et al.^14^	301	5 years	DLBCL first line I‐IV 60–80	R‐CHOP	MTV + PS	tMTV using 41% SUVmax Cut‐off 220 cm^3^ PS ≥2	ROC‐AUC analysis X‐tile analysis	4 years PFS 82% versus 63% versus 41% and 4 years OS 94% versus 79% versus 59% for pts with 0, 1 or 2 risk factors (*p* = 0.0001)	
	REMARC								

Abbreviations: ASCT, autologous stem cell transplant; DLBCL, diffuse large B‐cell lymphoma; MH, metabolic heterogeneity; OS, overall survival; PFS, progression‐free‐survival; PMBCL, primary mediastinal B‐cell lymphoma; R‐CHOP, rituximab, cyclophosphamide, doxorubicin, vincristine and prednisone; sTARC, serum thymus and activation regulated chemokine; SUV, standardized uptake value; tMTV, total metabolic tumor volume.

A model including baseline tMTV ≥220 cm^3^ (using 41% of the maximum SUV to delineate tumors) and performance status (PS) ≥2 was also developed in a population of patients aged 60–80 with DLBCL in the REMARC study treated with R‐CHOP.^14^ These two risk factors were the only independent variables associated with 4‐year OS from tMTV, IPI, NCCN‐IPI, B2 microglobulin, albumin and treatment arm (maintenance lenalidomide vs. placebo). The MTV/PS model with the specified cut‐offs outperformed the IPI for prediction of PFS and OS, with 4‐year OS of 94% versus 79% versus 59% for patients with 0, 1 or 2 risk factors respectively. The same model validated in two external clinical trials (GOYA and PETAL *n* = 1825) and real world (RW) data (*n* = 349) that included younger adults.^54^ Different measurement methods were used in the two clinical trials so the COMBAT method discussed above^42^ was used to translate values for tMTV obtained using the liver threshold in the GOYA trial to those using the 41% threshold in the PETAL trial and RW data.

Heterogeneity of FDG uptake was reported to be an independent predictor from TLG for PFS in 103 patients with PMBCL from the IELSG26 study.^19^ However heterogeneity measured in the hottest single lesion in patients with DLBCL was a less powerful predictor than tMTV, although heterogeneity was associated with OS and PFS in the subset of patients with high tMTV in a cohort of patients from the SAKK 38/07 study (test set *n* = 141, validation set *n* = 113).^20^

**FIGURE 1:**
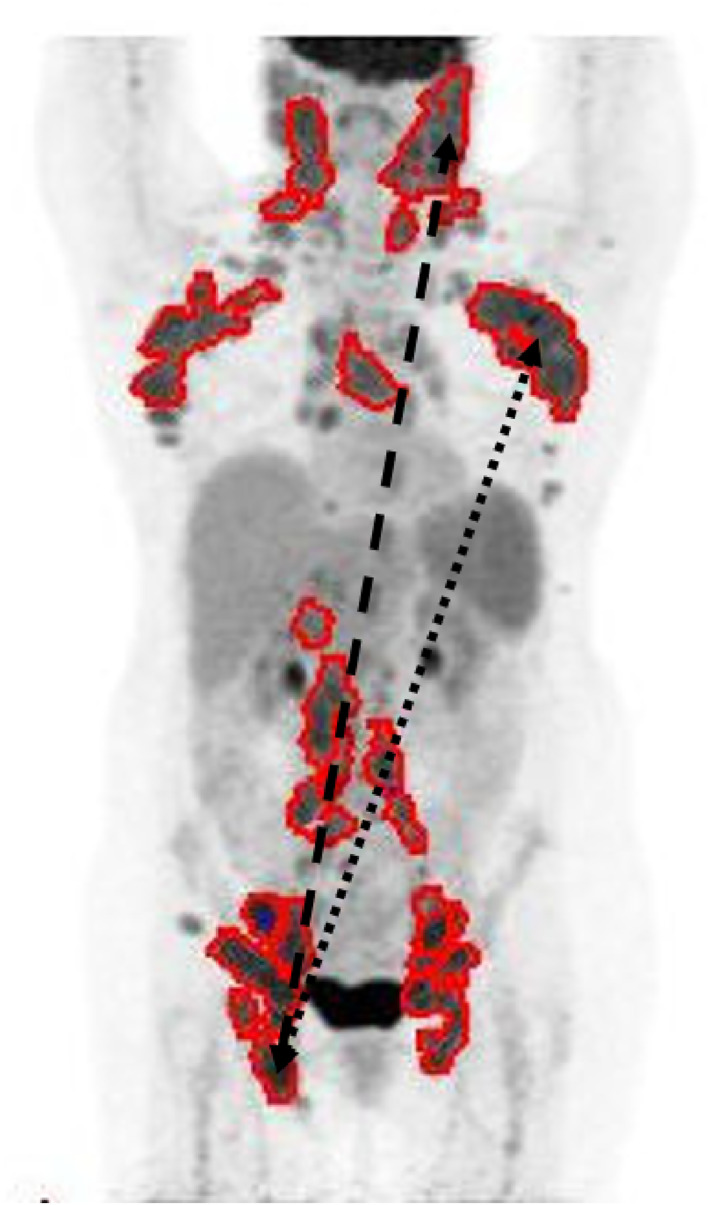
Maximum intensity projection image of a PET‐CT scan of a patient with multiple nodal groups involved by lymphoma at baseline. tMTV is shown in red and measures 693 cm^3^. Dmax is measured as the maximum distance between the centroids of the left neck nodal mass and the right femoral nodes and equals 675 mm (dashed line). Dmax bulk is measured as the maximum distance between the centroids of the left axillary nodal mass, which has the largest nodal metabolic volume and the right femoral nodes and equals 555 mm (dotted line). PET‐CT, positron emission tomography‐computed tomography; tMTV, total metabolic tumor volume.

Disease dissemination markers appear to be more promising biomarkers that are independent from tMTV and provide information beyond that afforded by imaging stage (Figure 1). This was first reported in patients with DLBCL by Cottereau et al.^21^, ^22^, ^55^ The simplest of the measurements reported was the largest distance between the center of two lesions in the body (Dmax) adjusted for body surface area (SDmax) but others such as the distance between the bulkiest lesion and the lesion furthest away (Dmaxbulk) as well as measurements of lesional spread, whereby distance between lesions are summed were also prognostic^21^ (Figure 1). In HL, Dmax was reported to be an independent predictor of PFS from baseline tMTV and clinical risk factors, adjusted for radiotherapy and treatment intensification^53^ including the subgroup who achieved early complete metabolic response (CMR). In the report by Durmo et al.,^53^ genes linked to known prognostic markers in the tumor microenvironment (e.g., CD20, FOXP3 and CD68) and cell populations controlling the immune environment were differentially expressed in patients with high versus low Dmax, providing a potential biological explanation.

The relationship of the IPI and its components, bulk disease and 490 radiomic features including disease dissemination were explored in 317 patients with DLBCL from the HOVON‐84 study, treated with R‐CHOP.^8^ The optimal radiomic model comprised the “conventional” PET metrics of tMTV4, SUVpeak (SUV in the hottest 1 cm^3^ of tumor) and Dmaxbulk in the patient. This simple radiomic model performed better than one using more complex radiomic features, including textural analysis of the hottest or the largest lesion. The simple radiomic model was significantly better than tMTV alone for prediction of 2‐year time‐to‐progression (TTP), confirming the important role that tumor burden and dissemination features could play in prediction of response. The radiomic model combined with clinical risk factors (IPI components and bulk >10 cm) were significantly better than the IPI and the clinical prediction model on their own. This work suggests that patient level rather than individual lesion analysis may be more informative in DLBCL and that radiomic analysis provides added value to standard prognostic clinical scores.

The same group subsequently reported that combining MYC status with patient level radiomic features (MTV, SUV metrics and dissemination features) in 323 patients from three clinical trials in aggressive B cell lymphoma improved the positive predictive value (PPV) compared with the IPI and IPI combined with MYC status, again supporting a role for radiomic analysis.^4^ The PPV for 2‐year TTP was 29.6% using IPI alone, 40.4% using IPI and MYC status and 50% using MYC status and radiomic features.^4^ Table 1 summarizes relevant studies reporting baseline radiomic analysis in clinical trials.

## 3 RESPONSE PREDICTION DURING TREATMENT

The simplest quantitative feature used in response prediction is the maximum SUV of residual lesions compared to reference regions of the liver and mediastinal blood pool, usually combined with visual interpretation in the widely adopted Deauville criteria.^56^ The change in SUV in DLBCL (delta SUV) in interim PET after 2 or 4 cycles of immunochemotherapy has been reported to improve the PPV whilst maintaining a high negative predictive value in DLBCL compared to Deauville criteria^57^, ^58^, ^59^ using a cut off of 66% at cycle 2% and 70% at cycle 4 of immunochemotherapy. Response adaptation using the delta SUV has been successfully applied in DLBCL.^57^, ^59^ The Deauville score^50^, ^60^ and change in SUV 1 month post CAR‐T cell therapy^61^ has also been reported to be predictive of OS.

A new concept of “near CMR” with >90% reduction in MTV has been suggested as a more sensitive method of response to checkpoint inhibitors than CMR, given either sequentially or in combination with doxorubicin, vinblastine and darcarbazine (AVD) chemotherapy in first‐line treatment of patients with HL.^62^, ^63^

The combination of emerging blood biomarkers as well as established risk factors with early PET scans, which may provide complementary information to monitor response is appealing. Kurtz et al.^36^ demonstrated that early molecular response after one or two cycles of treatment with circulating tumor DNA (ctDNA) in 217 patients with DLBCL and interim PET (in the subset of 66 patients who had early PET scans) were independent predictors of PFS and OS. Kurtz et al. later developed a model incorporating IPI, the cell of origin and interim PET in a small sample of 49 patients with baseline and 35 patients with interim results and tested this in a “validation” set of 132 patients.^5^ The model outperformed all the individual factors including IPI for the prediction of 2‐year event‐ free‐survival (EFS). The predicted and observed 2‐year EFS of the baseline risk were within 1% (95% confidence interval −3 to +4%) of one another and the predicted and observed EFS of the intra‐treatment risk at any time from 12 to 36 months were within 5%. Although the PET analysis was based on visual not “radiomic” analysis, this was one of the first publications to explore the concept of a dynamic risk score that could be updated during the course of treatment using established clinical risk factors, ctDNA and early PET scans to identify patients who could benefit from testing of novel treatment approaches.

In another report exploring baseline and intra‐treatment blood biomarkers and PET scans in HL,^27^ baseline serum thymus and activation regulated chemokine (sTARC), baseline SUV peak (the “hottest” 1 cm^3^ of tumor) and sTARC after one cycle 1 of brentuximab vedotin (BV)‐dexamethasone, cytarabine, cisplatin (DHAP) and SUVpeak prior to autologous stem cell transplant were shown to predict response in 65 patients with relapsed/refractory (R/R) disease in the transplant BRAVE study, where tMTV had low prognostic value.

## 4 WHEN WILL RADIOMIC ANALYSIS BE READY FOR PRIME TIME?

The most important prognostic features of tMTV and disease dissemination can be measured using currently available software. Progress is being made to standardize these measurements with international consensus,^28^ as was previously achieved for assessment of response with the Deauville criteria. Existing models in DLBCL incorporating (i) age, stage and MTV^35^ and (ii) PS and MTV^54^ can be applied to measure prognosis in the clinic now for patients treated with R‐CHOP without unduly adding to the length of clinical reporting. The prognostic value of these models will need to be tested in patients treated with polatuzumab vedotin, rituximab, cyclophosphamide, doxorubicin, prednisolone (R‐CHP),^64^ which is rapidly becoming an alternative standard approach.^65^ Newer algorithms incorporating artificial intelligence^40^ are likely to allow even faster and reproducible measurement of tMTV.

Disease dissemination will likely provide added prognostic value to measurement of tumor burden.^21^

The application of tMTV as a biomarker for risk and response adaptation is already being tested in a clinical trial, the “RAFTING” trial in patients with HL in combination with ctDNA (https://clinicaltrials.gov/ct2/show/NCT04866654).

It is clear that evaluation of radiomic features, molecular markers and ctDNA at baseline and during treatment should be factored into current clinical trial designs using appropriate statistical models to explore the relationship of continuous variables with patient outcomes.^35^ Promising radiomic and circulating blood biomarkers should be assessed with established clinical risk factors in clinical decision models. Prospective collection of data and retrospective analysis of curated imaging data from clinical trials will enable the development of baseline and dynamic risk scores that could further advance the field to facilitate testing of novel treatments and personalized therapy in aggressive lymphomas.

## CONFLICT OF INTEREST STATEMENT

The author declares no conflicts of interest.
